# Support for young people who are distressed by hearing voices: protocol for an uncontrolled feasibility evaluation of a psychological intervention package delivered within secondary schools (the ECHOES study)

**DOI:** 10.1186/s40814-025-01611-x

**Published:** 2025-04-04

**Authors:** Mark Hayward, Mary John, Sarah Parry, Anna-Marie Bibby-Jones, Faith Orchard, Fiona Malpass, Clare Dixon, Akira Naito

**Affiliations:** 1 University of Sussex, Brighton Bn1 9rh and Research and Development Department, Sussex Partnership NHS Foundation Trust, Hove, BN3 7HZ UK; 2 Research & Development Department, Sussex Partnership NHS Foundation Trust, Hove, BN3 7HZ UK; 3 Young People’S Mental Health Research Centre, Pennine Care NHS Foundation Trust, 225 Old Street, Lancashire, Ashton-Under-Lyne OL6 7SR UK; 4 School of Psychology, University of Sussex, Brighton, BN1 9RH UK; 5 School of Psychology, University of East Anglia, Norwich, NR4 7TJ UK; 6 Voice Collective, Barnes House, 9-15 Camden Road, London, Mind in Camden NW1 9LQ UK

**Keywords:** Voice hearing, Young people, Coping, Schools, Feasibility

## Abstract

**Background:**

Hearing voices is a common experience for young people and can cause significant distress. There are no evidence-based psychological interventions for distressing voices in young people, although a focus on coping strategies has been suggested as a useful approach. We have developed and evaluated a brief 1:1 coping intervention for young people who hear distressing voices. This intervention has been successfully piloted within Child and Adolescent Mental Health Services (CAMHS), together with a psychoeducational workshop for parents. The 1:1 intervention and the workshop will be combined into an intervention package and offered within schools to maximise accessibility.

**Methods:**

This study will be an uncontrolled feasibility evaluation addressing the following questions: is the intervention package acceptable to young people, those who support them, and staff and practitioners within secondary schools? What is the optimum content, structure, and duration for the intervention package? Is delivery of the intervention package feasible for the practitioners and what are their requirements for training and supervision? What tools can be used to evaluate the impact of the intervention package?

The study will be guided by the MRC Framework for the development of complex interventions and consist of an iterative process over four phases: phase 1—adaptation of the intervention package with young people, parents, and school staff; phase 2—delivery of the intervention package through Mental Health Support Teams to students, supporters who have been nominated by the students and school staff; phase 3—analysis of quantitative and qualitative data collected from participants and practitioners; phase 4—further adaptation of the intervention package with young people, parents and school staff.

**Discussion:**

If the findings from the study suggest that a future trial is warranted, a feasibility Randomised Controlled Trial will be designed to establish the parameters for a definitive trial.

**Trial registration:**

Current Controlled Trials ISRCTN registration number: 16395888. Registered on 11 January 2024. 10.1186/ISRCTN16395888.

**Supplementary Information:**

The online version contains supplementary material available at 10.1186/s40814-025-01611-x.

## Background

The experience of hearing voices (also known as auditory hallucinations) is a common experience for young people, with prevalence rates estimated at 12% [1]. This experience has been associated with an increased risk for suicidal ideation, plans and attempts [2], and self-harm [3]. When voice hearing persists in young people, this can be indicative of severe mental health problems in the future [4], including psychosis [5]. Young people have reported feeling lost, not listened to, and found it difficult to obtain useful information when seeking help for voice hearing within mental health services [6]. They also report many barriers to disclosure [7]. The individuals from whom young people may seek help have also reported a lack of confidence to talk about voice-hearing and offering advice (e.g. mental health practitioners [8], parents [9], and teachers [10]).

There are currently no psychological interventions that have an evidence base specifically for the treatment of distressing voices for young people. A focus on coping strategies has been suggested by young people as a useful approach to intervention [11]. Within the Sussex Voices Clinic (https://www.sussexpartnership.nhs.uk/sussex-voices-clinic) we have developed a brief coping intervention for the treatment of distressing voices in young people. This intervention was piloted within CAMHS [12] and generated pre-post reductions in voice-related distress. Feedback from young people was suggestive of the value of a space to talk about voices (e.g. ‘[the therapist] was easy to talk to and just listened without judging’) and a focus on coping strategies (e.g. ‘it taught me lots of different ways to block out the voice, move forward and not dwell on things’) [12]. We also piloted a psychoeducational online workshop that was offered to parents/carers and outcomes suggested that participants were more confident to offer useful advice about voice-hearing to a young person [13].

We will combine the brief coping intervention and the psychoeducational workshops to form an intervention package to be delivered within schools where early intervention and accessibility can be maximised. This will be facilitated by the Mental Health Support Teams in Schools (MHST) initiative, a national initiative whereby trained mental health practitioners are located within schools to address the psychological wellbeing of young people [14].

The study will address the feasibility of offering an intervention package within secondary schools to young people who hear distressing voices and the people who support them.

## Methods

### Aim and research questions

The study will be an uncontrolled feasibility evaluation guided by the MRC Framework for the development of complex interventions [15] and consist of an iterative process that will proceed over four phases: phase 1—development of the intervention package; phase 2—delivery of the intervention package; phase 3—collection and analysis of quantitative and qualitative data; and phase 4—co-evaluation of the intervention package.

The following questions will be explored as the intervention package is ‘tested and refined on a small scale’ [16]

- Is the intervention package acceptable to young people, those who support them, and staff and practitioners within secondary schools?
- What is the optimum content, structure, and duration of the intervention package?
- Is delivery of the intervention package feasible for the MHST practitioners and what are their requirements for training and supervision?
- What tools can be used to evaluate the impact of the intervention package?
- Can participants be retained within the study and offer full datasets?

### Setting

The study will be conducted within the secondary schools that are part of the Mental Health Support Teams (MHST) in Schools initiative in West Sussex—called Thought-Full. This initiative is co-ordinated jointly by Sussex Partnership NHS Foundation Trust and West Sussex County Council. There are currently 13 secondary schools that have become established within Thought-Full (others are in set-up), each of which receives support from Education Mental Health Practitioners (EMHP) and Senior Mental Health Practitioners (SMHP) to deliver evidence-based low-intensity psychological interventions, develop the whole school approach to mental health and well-being and help students to get the right support.

### Inclusion and exclusion criteria

Volunteers (phases 1 and 4—we refer to these participants as ‘volunteers’ to differentiate them from the ‘participants’ who will take part in phases 2 and 3):

- Students will be students within years 7–11 (ages ranging from 11 to 16 years) at one of the schools served by Thought-Full. There will be no criterion related to personal experience of hearing voices and/or mental health problems. The students will need to be willing and able to provide written informed assent. Assent to approach the student’s parent/carer will be required for all students. The parent/carer of the students will need to give written informed consent.
- Parents will be parents of students within years 7–11 at one of the schools served by Thought-Full. There will be no criterion related to the student or the parent having personal experience of hearing voices and/or mental health problems. It will be permitted (but not required) for the parent’s child to be a volunteer within the student’s group.
- School staff will be permanent members of staff at one of the schools served by Thought-Full. There will be no criterion related to the staff having pastoral responsibilities within the school.

Participants (students—phases 2 and 3):

- Attending one of the schools selected for participation in phase 2
- Within years 7–11 of the selected schools
- Willing and able to provide written, informed assent. Assent to approach the student’s parent/carer will be required for all students. The parent/carer of the student will need to give written informed consent.
- Reporting a current voice-hearing experience—operationalised by participants having a score of at least 1 on item 1 on the Hamilton Voices Questionnaire (HPSVQ) [17]—indicating that the participant has experienced at least one episode of voice-hearing in the past week
- Reporting that voice-hearing experiences are causing distress—operationalised by a score of at least 8 (range from 0 to 16) on the negative impact scale of the HPSVQ
- Not a volunteer in this study

Participants (Plus-1s [a trusted adult selected by the student]—phases 2 and 3):

- Nominated by one of the student participants
- Aged 16 years or over
- Willing and able to provide written informed consent.
- Not a volunteer in this study
- Not a student in secondary education.

Participants (school staff—phases 2 and 3).

- A member of permanent staff at one of the selected schools
- Willing and able to provide written informed consent
- Not a volunteer in this study

### Intervention protocol

The intervention package to be evaluated within the study will include two components (a 1:1 coping intervention for students and an online psychoeducational workshop for Plus-1s and school staff) as follows:

1. Coping intervention (to be offered to student participants)—approximately four sessions (offered weekly) of individual therapy (each with a maximum duration of 60 min), guided by a workbook:
  Session 1: a semi-structured interview is used to identify the antecedents to voice activity (e.g. striving to achieve high standards, night-time, feeling anxious/low in mood/angry) and the young person’s emotional and behavioural responses to the voices (self-harm, spending time with friends, talking back to voices, etc.). The ability of these responses to facilitate coping or increase distress is considered. Discussions also explore the times/places when voices are not active. These discussions stimulate a process of identifying coping strategies and evaluating their effectiveness.
  Session 2: an existing coping strategy is collaboratively selected and considered in detail. Discussions focus on how the strategy could be modified and used differently (e.g. more or less often). A plan is agreed to implement the strategy between sessions.
  Session 3: implementation of the modified coping strategy is reviewed. Discussions focus on the enablers and barriers to implementation, and the effectiveness of the strategy. This strategy could be further modified to enhance effectiveness, or another strategy could be selected and modified. A plan is agreed to implement the strategy between sessions.
  Session 4: implementation of the modified coping strategy is reviewed, and any required modifications are agreed upon. Plans are discussed for continued implementation post-intervention. Discussions explore any learning from the intervention in relation to both self and voices, and the implications of this learning for living well with voices. Any needs for further intervention are discussed.

During term time, the coping intervention will be delivered face-to-face within schools to student participants by EMHPs who will be trained and supervised by Mark Hayward and Mary John. Outside of term time, the intervention may be offered face-to-face in community settings or remotely via secure online platforms.

2) Psychoeducational workshop (to be offered separately to Plus-1s and school staff)—a 2-h workshop exploring facts and fiction about voice hearing, the principles of the coping intervention, and tips for talking about voice-hearing:
  Facts and fiction—‘fact or fiction’ statements about voice hearing are considered. The statements encourage attendees to reflect on their existing knowledge and dispel common misconceptions. The knowledge imparted by the statements is reinforced by statistics on the prevalence of voice-hearing across the lifespan.
  Coping intervention—attendees are introduced to the principles of the coping intervention that is being received by the student participants. Discussions focus on what happens before voices (triggers), what happens after voices (helpful and unhelpful responses) and when are voices not around? A case study of a fictional young person is used interactively to apply the principles to everyday experience.
  Tips for talking about voices—the final part of the workshop covers tips for talking about voices with a young person. These tips are taken from the chapter for carers within the CBT self-help book ‘Overcoming Distressing Voices’ [18]. Emphasis is placed upon acceptance, being non-judgmental, building warmth and space, and being curious.

The psychoeducational workshops will be co-facilitated by Mark Hayward and an SMHP. The SMHPs will be trained and supervised by Mark Hayward and Mary John. The method of delivery and timing of the workshops will be negotiated with the participants in relation to their availability. There will be a maximum of 12 participants in each workshop.

All participants can receive and engage with other interventions during their participation in the study.

### Outcomes

#### Feasibility assessment

The assessment of feasibility for this study will address the following questions:

Is it acceptable to young people, those who support them, and staff and practitioners within secondary schools?

- The number of students, Plus 1s, and teaching staff who offer to become participants
- Amongst the students, Plus 1s, and teaching staff offering to be participants, the number and proportion who are found to be eligible
- The number and proportion of consenting participants who complete an intervention(s) and offer full data sets.

What is the optimum content, structure, and duration?

- Feedback from volunteers and participants during phases 3 and 4

Is delivery feasible for the MHST practitioners and what are their requirements for training and supervision?

- Feedback from practitioners during phase 3.

What tools can be used to evaluate impact?

- Feedback from volunteers during phase 4.

### Clinical outcome measures

The process of selecting outcome measures for use in phase 2 will be guided by the consultations with volunteers in phase 1. We will offer the volunteers a range of candidate measures to inform their discussions, e.g. the Hamilton Voices Questionnaire [17] (used within our evaluation of the coping intervention within CAMHS) [12]; the Manchester Voices Inventory for Children [19] (developed by Sarah Parry to assess the voice-hearing experiences of young people), the Strengths and Difficulties Questionnaire (routinely used within the MHST) [20], the Revised Child Anxiety and Depression Scale (routinely used within the MHST) [21] and the Attitudes and Beliefs Towards Voice Hearing measure (used within our evaluation of the psychoeducational workshops) [13].

### Sample size

The sample size for the qualitative interviews in phase 3 was based on guidance on reaching meaning saturation in qualitative analysis [22]. We used the upper limit of *N* = 24 interviews per group to increase the likelihood of identifying and understanding all themes emerging from our analysis. Additionally, we accounted for a 25% attrition rate, as reported in our previous studies [12, 13]. This results in a sample size of 32 per group.

The most recent literature for designing feasibility and pilot studies recommends sample sizes between 28 and 35 for a single study arm [23, 24]. Based on these recommendations and a recent sample size review [25], a sample size of 30 is sufficient data to provide the key information needed to design a future RCT. This sample size will also be sufficient for calculating the 95% confidence level for the target 75% retention rate with a margin of error of ± 15%. Additionally, it will allow for conducting within-group descriptive summaries of the pre- and post-workshop quantitative measures to be identified in phase 1.

### Recruitment

#### Phase 1

Volunteers will be recruited from the secondary schools within Thought-Full. The opportunity to volunteer will be advertised through the usual promotional channels used by Thought-Full. Approximately 12 volunteers will be recruited for each of 3 groups: students, parents/carers, and school staff. The focus group for students may be split into smaller groups, if this is indicated by the age range of the students, e.g. students from years 7 and 8, or 10 and 11 may have a separate group).

All those who express an interest in volunteering will be sent a Participant Information Sheet (PIS) and have at least 48 h to review its contents. Prospective volunteers will be invited to a meeting with a Research Assistant (RA) where informed assent/consent will be given and eligibility confirmed. Thereafter, the volunteers will be invited to attend the appropriate volunteer meeting. These meetings will last for a maximum of 90 min and the timing will be negotiated (e.g. the taking of breaks). The method/location for delivery of the meetings (online and/or face-to-face) will be influenced by the geographical spread of the volunteers.

Each group of volunteers will have a separate meeting in the first instance, as the power differentials between the groups may inhibit communication and participation. The initial task for each group of volunteers will include (1) reviewing the content and materials associated with the coping intervention and the psychoeducational workshop and developing an intervention package for delivery within a school environment; and (2) selecting tools from a range of options for the assessment of outcomes. A fourth meeting will bring together volunteers from the three groups and explore any differences in views about the development of the intervention package. In the event of different views not being reconciled, these views will be taken to the Lived Experience Advisory Panel (LEAP) which will make a final decision. The volunteers in the student and parent/carer groups will each be reimbursed with a £25 voucher after attending each meeting. The volunteers in the school staff group will not be reimbursed as they will be attending in paid time. See Table 1 for the volunteer journey through the study.

**Table 1:** Volunteer journey (phases 1 and 4)

Week^a^	Event	Who
0	Advertise volunteering opportunities through approved channels and processes	Researcher
0	Potential volunteer receives PIS from an approved source	
1	Contact is made with potential volunteers to set up the eligibility and consent meeting	Researcher
1	Volunteer can ask questions, complete the eligibility assessment, and complete the consent form	Researcher
2	Contact will be made to arrange the stakeholder meetings	Researcher
3–12	Phase 1 stakeholder meetings will be conducted	Researcher
13–56	Occasional email/text/phone contact to maintain engagement	Researcher
57–69	Phase 4 stakeholder meetings	Researcher

^a^Relates to the duration of the volunteer’s time in the study, not to the study timeline

#### Phase 2

Participants will be recruited from a sub-set of selected secondary schools within Thought-Full. Decisions about the schools to be selected will be guided by the capacity of schools (e.g. availability of Thought-Full practitioners) and the needs of the local population (e.g. indices of deprivation, SEN data, ethnicity data, and referrals to established emotional well-being community services and CAMHS). Students will be referred through the usual Thought-Full processes via self-referral or via parent/school staff; Plus-1s will be nominated by referred students and the student will provide the Plus-1 with a PIS and ask them to contact the research team if they wish to participate; and school staff will self-refer. The opportunity to participate will be advertised through the usual promotional channels used by Thought-full. Approximately 32 participants will be recruited for each of the three groups: students, Plus-1s, and school staff. All those who express an interest in participating will be sent a PIS and have at least 48 h to review its contents. Each prospective participant will be invited to a meeting with the RA where informed assent/consent will be given, eligibility will be confirmed, and baseline measures completed. Thereafter, each participant will be offered the appropriate element of the intervention package; the 1:1 coping intervention for students; the psychoeducation workshop for the Plus-1s; and a separate psychoeducation workshop for school staff. See Table 2 for the participant’s journey through the study.

**Table 2:** Participant journey (phases 2 and 3)

Week^a^	Event	Who
0	Advertise research participation opportunity through approved channels and processes	Researcher
0	Potential participant receives PIS from approved source	
0	Referral made where the potential participant is interested in participation	MHST or Teaching staff
1	Contact is made with potential participant to set up the eligibility and consent meeting	Researcher
1	Participant can ask questions, complete the eligibility assessment, complete the consent form and the baseline assessment	Researcher
2	Contact will be made to arrange the intervention session(s)	Researcher or MHST staff
3–12	Intervention session(s) will be delivered	MHST staff
13–14	Final assessment meetings	Researcher

^a^Relates to the duration of the participant’s time in the study, not to the study timeline

#### Phase 3

After the completion of the intervention, participants will be invited to a final assessment meeting where measures will be completed, and feedback will be sought. The meetings will be facilitated by Sarah Parry and the RA and will be offered on a 1:1 basis for student participants and as separate focus groups for Plus-1 participants and school staff participants. The meetings will take place either in person or remotely, depending on the location, availability, and preferences of the participants. The EMHPs and SMHPs who deliver the intervention package will be invited to a separate focus group to reflect upon their experience and will be asked to provide an anonymously written reflection, which will ensure we account for biases inherent in face-to-face qualitative work where the designers of the intervention also evaluate the process. The student participants and Plus-1 participants will be reimbursed with a £20 voucher after attending each of the assessment meetings (baseline assessment and final assessment). The school staff participants will not be reimbursed as they will be attending assessment meetings in paid time.

#### Phase 4

The three groups of volunteers who worked on phase 1 will be invited back to revise the intervention package in light of the learning from phases 2 and 3. The process will be the same as phase 1, whereby the groups will meet separately before coming together for a final meeting. Any differences of view that cannot be reconciled will be taken to the LEAP for a final decision. We will maintain contact with the volunteers during the months between phases 1 and 4 to facilitate ongoing engagement. This contact will take the form of regular updates and newsletters. The volunteers in the student and parent groups will each be reimbursed with a £25 voucher after attending each meeting. The participants in the school staff group will not be reimbursed as they will be attending in paid time.

### Data collection, entering, coding, and checking process

The online Qualtrics™ data collection tool will be set up by the RAs and checked by the statistician. Each participant will have a unique code which will be used in Qualtrics™ and on paper assessment booklets instead of the participant’s name.

Demographics and clinical measures data will be collected by RAs using Qualtrics™ as the primary tool. This will be accessed using a wifi connection on their study/Trust laptop. In the event of wifi/Qualtrics not being available, RAs will complete paper assessment booklets containing the questionnaires during the assessment with the participant. Any mistakes on the form should be cleanly crossed out, initialled, dated, and rewritten (all in black ink). The error should not be overwritten. Correction fluid should not be used. The RA will transfer the data from the paper booklet to Qualtrics™ as soon as possible, i.e. when they reach their workbase. RAs must follow individual instructions for each of the clinical measures.

RAs will quality check a 10% sample of assessments recorded on paper booklets and then enter it into Qualtrics when the data is downloaded into Excel. The statistician will identify the sample that needs quality checking.

All data downloaded from Qualtrics™ will be stored on the Trust’s network in a password-protected file. Any data shared must be password protected.

Eligibility screening and monitoring data will be recorded on a password-protected Excel spreadsheet by the RA and checked by Mark Hayward.

Feasibility analysis and analysis of the clinical outcomes will be carried out by the statistician.

The qualitative data will be generated through the use of semi-structured interviews that will be audio-recorded using an encrypted audio device. The audio-recordings will be uploaded to Sharepoint, a secure web-based collaborative platform, from which they will be downloaded and stored on the secure drive of the R&D Department at Sussex Partnership NHS Foundation Trust. The audio recordings will be transcribed verbatim into Word documents, after which the audio files will be deleted.

The analysis of the qualitative data will be co-ordinated by Sarah Parry.

### Missing data policy

We aim to minimise missing data at the point of collection.

In Qualtrics™ a flag for missing data will be added by the RA at set-up to double-check if any missed questions are intentional. If using the paper-based booklets, at the end of the assessment the RA will double-check that all questions have been answered.

At the point of coding, the following missing data values will be used.

666 = dropped out of the study.

888 = not applicable.

999 = question not answered.

1/1/999 = missing date.

At the point of analysis: data will be summarised to look at patterns of missingness.

### Data analysis

Qualitative—phase 1: a consensus amongst the groups as to the most important content and communication style for the intervention package within a school setting will be achieved through the process of concept mapping [26]. In their groups, the volunteers will identify important features of the intervention [27]. Through a process of facilitated negotiation and conversation, the groups will develop consensus maps (construct maps, akin to mind maps in presentation) of the key features they think should be included in the intervention package.

Quantitative—phases 2 and 3: recruitment and retention rates will be calculated for the data generated and used to assess the feasibility of delivering the intervention package and its acceptability. The level of study diversity and inclusion will be assessed through a summary of the demographics using descriptive statistics as appropriate. Quantitative measures included in the evaluation tools will also be summarised using descriptive statistics. Quantitative measurements collected pre- and post-intervention will be used to indicate average levels of change with associated 95% Confidence Intervals, but no hypothesis testing will be carried out. This information will be for indicative purposes only. Levels of data completeness will be summarised.

Qualitative—phase 3: the final assessments are expected to last between 40 and 90 min, depending on how much people would like to say. Discussions about feedback will be informed by a Topic Guide and will follow a flexible structure to enable the participants to lead the conversation. Questions and prompts will be asked by the researcher to encourage reflection on experience and elicit information to inform the ongoing development of the intervention materials.

Anonymised verbatim transcripts from participants will be analysed using the Framework Method [28], commonly employed in the evaluation of studies of complex interventions in health research. According to the priorities of the recommendations from the MRC [15], this part of the analysis will attend specifically to generating a nuanced understanding of the impact of the intervention for individuals, assessing its value relative to the reasonably limited resources required to deliver it, and theorising how it works for individuals in the school environment. Through data provided by students, Plus-1s, and school staff, we will begin to understand how the intervention may contribute, or not, towards systemic changes within the school culture in relation to mental health, stigma, and decision-making. To maintain careful attention to the idiographic nature of participants’ reflections, leading to an in-depth inductive analysis, we will follow the seven steps of the Framework Method.

### Safety reporting and monitoring

Any unfavourable and unintended sign, symptom, or illness that develops or worsens during the period of the study will be classified as an Adverse Event (AE), whether or not it is considered to be related to the study intervention. AEs will include an exacerbation of a pre-existing illness; an increase in the frequency or intensity of a pre-existing episodic event or condition; a condition that is detected after study intervention administration; and continuous persistent disease or a symptom present at baseline that worsens following administration of the study intervention—and may be expected or unexpected. Serious adverse events (SAEs) are those considered to be life-threatening, resulting in death, requiring inpatient hospitalisation, or prolongation of existing hospitalisation, resulting in significant or persistent incapacity/disability or a birth defect or congenital abnormality. The number (events and individuals) and nature of all events (AEs and SAEs) reported to members of the research team will be recorded. The period for AE reporting will be following the signing of the study consent form until the completion of phase 4 (volunteers) or the completion of the final assessment within phase 3 (participants). All AEs will be recorded and reviewed by the Chief Investigator. If an AE is considered to be serious (an SAE), it will be reviewed for causality and expectedness by the Chief Investigator and an independent rater. SAEs will be reported to the NHS Research Ethics Committee, as appropriate.

The study is covered by the indemnity insurance of the NHS and West Sussex County Council.

### Ethics approval and consent to participate

The study is sponsored by Sussex Partnership NHS Foundation Trust. NHS Research Ethics Committee (North West—Preston, reference number 23/NW/0334), Health Research Authority, and local Research Governance approval was granted before the commencement of the study (study protocol—version 2, dated 04/11/23 [see Table 3 for approved amendments to protocol]).

**Table 3:** Approved amendments to protocol

Amendment number	Protocol version	Details of change
1)	Version 2 (04.11.23)	• Addition of versions of consent forms for remote meetings
2)	Version 2 (04.11.23)	• Updating of Demographics Questionnaire to include additional protected characteristics
3)	Version 2 (04.11.23)	• Introduction of a measure to assess the ‘Goal Based Outcomes’ of students in Phase 2
4)	Version 2 (04.11.23)	• Introduction of additional measures (self-efficacy questionnaire and compassion to others questionnaire) to assess outcomes for Plus-1s and school staff • Easy Read Versions of assent/consent forms and participant information sheets for all participants in Phase 2

Assent and consent to take part in this study will be informed. Volunteers and participants who are students will be asked to provide assent to a parent/carer providing consent. All volunteers and participants will be given the PIS at least 48 h before meeting with a researcher to give consent. Furthermore, volunteers and participants will have the opportunity to ask questions about the research study before providing consent. The combination of the PIS and the opportunity to ask questions of a member of the research team will ensure that any consent given will be fully informed.

### Project management

The CI will chair monthly meetings of the research team where adherence to the research protocol will be checked, and problems will be identified and addressed. He will supervise the RAs who will coordinate the day-to-day administration of the study.

The Lead Governance Officer at Sussex Partnership NHS Foundation Trust will manage the study finances.

### Patient and public involvement

A Lived Experience Advisory Panel (LEAP) of young people with lived experience of hearing voices will be formed by Voice Collective from its existing networks. The LEAP will meet regularly to offer advice on the co-decision-making process, the recruitment and retention of participants, the co-evaluation of feedback, and the dissemination of findings. Young people will be remunerated for their time and input to the LEAP, and they will have ongoing support and mentoring from the Voice Collective team. Fiona Malpass and colleagues at Voice Collective will work to ensure that young people’s opinions and ideas are heard throughout the study, and will act as advocates if needed, but will also support the young people to feel empowered to share their thoughts themselves, where possible. Voice Collective will ensure that young people have multiple ways of expressing themselves and getting their thoughts across and will be mindful of inequalities and the potential privileging of certain views over others. Voice Collective will aim to support young people from backgrounds and identities that are often under-served by research processes, to be involved and feel heard.

The co-decision-making and co-evaluation processes within phases 1 and 4 will be guided by the ‘top tips’ for engaging young people as advisors in research [29], lessons learnt from attempts to co-produce with young people within education settings [30] and the NIHR guidance on co-producing a research project [31].

### Equality, diversity, and inclusion

This study will embrace a systemic approach by engaging with and including families, friends, school staff, and young people, irrespective of their experiences with mental health problems. This will avoid singling out those who hear voices that could otherwise lead to, or further perpetuate, stigmatising attitudes from others and act as a barrier to study participation.

We aim to engage a diverse range of young people, i.e. those who are from different ethnic minority groups, identify as Lesbian, Gay, Bisexual, Transgender, Queer or who use other terms (LGBTQ +), experience a learning disability or neurodiversity, are financially disadvantaged, or are from a deprived neighbourhood.

We will monitor the diversity of our volunteers and participants by collecting demographic data as appropriate, i.e. age, sex, gender, LGBTQ + status, ethnicity, and postcode for young people, with additional questions for adults, e.g. relationship status.

Barriers to participation may arise for families with multiple children or single-parent households having childcare needs and for people from economically disadvantaged backgrounds. We will therefore cover childcare costs and reimburse travel expenses associated with taking part in the study.

In our recruitment materials, we will actively state that we are looking for volunteers and participants from minoritised groups. The ability to make adaptations will be made clear, e.g. interpreters for those who do not speak English, and different formats for neurodiversity. With regard to written text, we will use plain English. The materials will also be translated into the three most common non-English languages spoken within the recruiting areas. Where imagery is used, we will incorporate a range of people reflecting diversity in ethnicity, physical ability, and age, for instance. We will consult with the LEAP on matters of EDI.

Voice Collective works with young people who by the nature of their voice-hearing experiences, are often alienated and marginalised. This often intersects with other aspects of their lives and identities, such as sexual orientation, gender identity, being from racialised communities, having multiple health or mental health diagnoses, etc.—which are also often sources of social stigma and marginalisation. We work to provide spaces of solidarity and allyship to young people, as well as giving platforms for the young people themselves to speak and influence issues that matter to them. We aim to work in ways that are culturally sensitive and consider accessibility needs as much as we can, including using caption technology in online meetings and BSL when possible/needed, ensuring that colours and fonts on materials are within recommended guidelines to be more readable for those with dyslexia and colour blindness, offering ‘easy read’ versions of materials, as well as with larger fonts, should they be needed. We build meaningful relationships with each young person and empower them to recognise their needs so that we can make sure we meet them in a way that the individual tells us would be helpful. These inclusive principles will guide our approach towards the facilitation of the LEAP.

We will carefully follow the NIHR INCLUDE roadmap and reflective questions to guide our decision-making and approach in relation to working inclusively. We will also use the reflective questions from the INCLUDE project to guide critical reflections and planning within the meetings of the LEAP throughout the study.

### Progression criteria

The success of the intervention package will be determined both quantitatively and qualitatively. Quantitative success will be achieved if at least 75% of the participants are recruited within each of the three groups within phase 2. Furthermore, at least 75% of these participants will offer full datasets. Qualitative success will be determined by the feedback offered by participants during their post-intervention interviews.

## Discussion

This study will evaluate the feasibility of offering an intervention package to students within secondary schools who are distressed by hearing voices and their supporters. Outputs will include lessons learnt from co-decision-making and co-evaluation processes within a school environment. Information will also be generated on the feasibility and acceptability of the intervention package. We will share our findings widely with the volunteers and participants, schools, voice-hearing communities, and MHST and CAMHS networks. We will work with the LEAP to ensure that information is pitched at an appropriate level, using creative ways to summarise and share findings.

If the findings from the study suggest that a future trial is warranted, a feasibility Randomised Controlled Trial will be designed to establish the parameters for a definitive trial.

## Supplementary Information

Supplementary Material 1. Assent Form (students in Phases 2 and 3).

Supplementary Material 2. Student Information Sheet (Phases 2 and 3)R1.

## Data Availability

Only anonymised data may be shared outside of the research team. A data-sharing agreement will be set up with individuals who are outside of the research team and who request to use the study data. The Chief Investigator can give approval for data-sharing requests. The possibility of data sharing will be made explicit to participants on the study consent form. The Participant’s personal details will not be shared with anyone outside of the research team. All data-sharing processes will adhere to the 2018 GDPR.

## Abbreviations

AE: Adverse event
CAMHS: Child and Adolescent Mental Health Services
CBT: Cognitive Behaviour Therapy
EDI: Equality, Diversity, and Inclusion
EMHP: Educational Mental Health Practitioner
HPSVQ: Hamilton Programme for Schizophrenia Voices Questionnaire
LEAP: Lived Experience Advisory Panel
LGBTQ +: Lesbian, Gay, Bisexual, Trans-gender, Queer and other terms
MHST: Mental Health Support Team
MRC: Medical Research Council
NIHR: National Institute for Health and Care Research
PIS: Participant Information Sheet
SAE: Serious Adverse Event
SMHL: Senior Mental Health Lead
SMHP: Senior Mental Health Practitioner
