# Supplementary material for: Support for young people who are distressed by hearing voices: protocol for an uncontrolled feasibility evaluation of a psychological intervention package delivered within secondary schools (the ECHOES study)
Source: Pilot Feasibility Stud. 2025 Apr 4;11:39. doi: 10.1186/s40814-025-01611-x (PMC11971772; doi:10.1186/s40814-025-01611-x)
Supplement: Supplementary file 1 — Supplementary Material 1. Assent Form (students in Phases 2 and 3). [file 40814_2025_1611_MOESM1_ESM.docx]

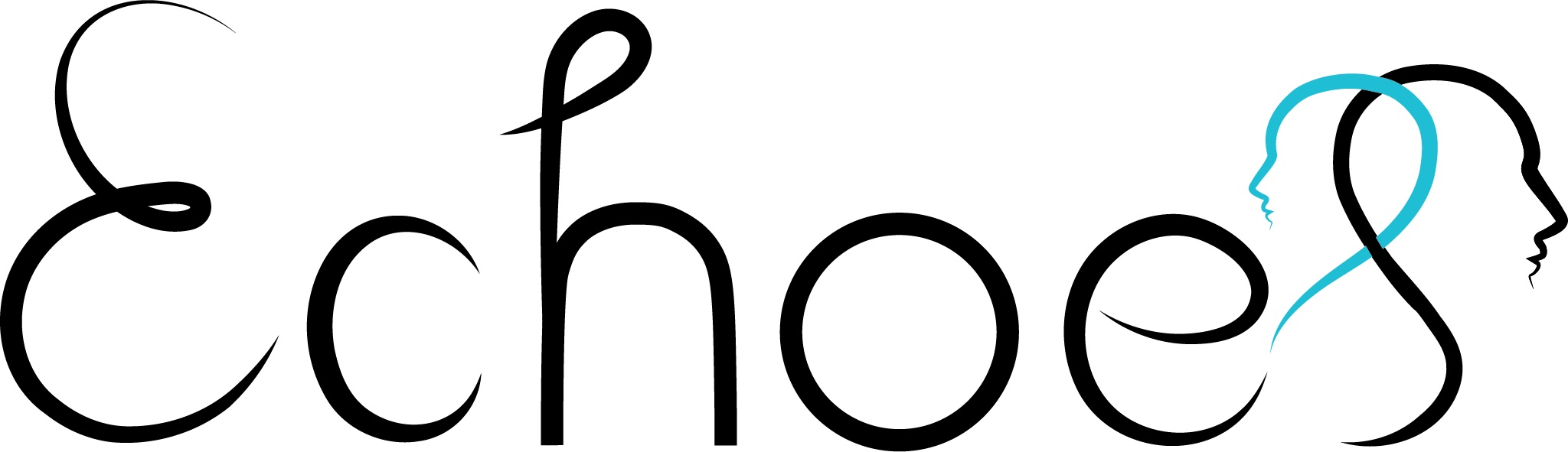


**ASSENT FORM**

For students participating in Phases 2 and 3

*(1 copy to participant; 1 copy to researcher; 1 copy to medical record)*

**Title of Study -** **Support for young people who are distressed by hearing voices: Preliminary evaluation of a psychological intervention package for delivery through Mental Health Support Teams within secondary schools (the ECHOES study)**

Principal Investigator: Professor Mark Hayward

***If you want to take part in this study, we need you and you parent/carer to agree. This document is a record of your agreement and also makes sure that you have been given all the information you need to make a decision. Please answer the questions and then sign your name if wish to take part.***

| Have you read (or had read to you) the information booklet about this research? | YES / NO |
| --- | --- |
| Has someone explained the research to you? | YES / NO |
| Have you asked any questions you want to? | YES / NO / NO QUESTIONS |
| Did you understand the answers? | YES / NO / NO QUESTIONS |
| Do you understand that it’s ok to stop taking part at any time, without giving a reason? | YES / NO |
| Are you happy for the feedback meeting to be audio recorded? | YES / NO |
| Are you happy to take part? | YES / NO |
| Are you happy for us to contact your parent(s)/carer(s) to request their permission for you to take part? | YES / NO |
| This item is optional:  I would like to receive a summary of the findings when the study is completed.  If you respond ‘Yes’ to this item, the research team will retain your contact details in order to provide the summary of findings. | YES/NO |

Name of researcher taking assent: ____________________

Signature: _________________________________________________________

Date: ____________________

Your name: Date:
