# Supplementary material for: Support for young people who are distressed by hearing voices: protocol for an uncontrolled feasibility evaluation of a psychological intervention package delivered within secondary schools (the ECHOES study)
Source: Pilot Feasibility Stud. 2025 Apr 4;11:39. doi: 10.1186/s40814-025-01611-x (PMC11971772; doi:10.1186/s40814-025-01611-x)
Supplement: Supplementary file 2 — Supplementary Material 2. Student Information Sheet (Phases 2 and 3)R1. [file 40814_2025_1611_MOESM2_ESM.docx]

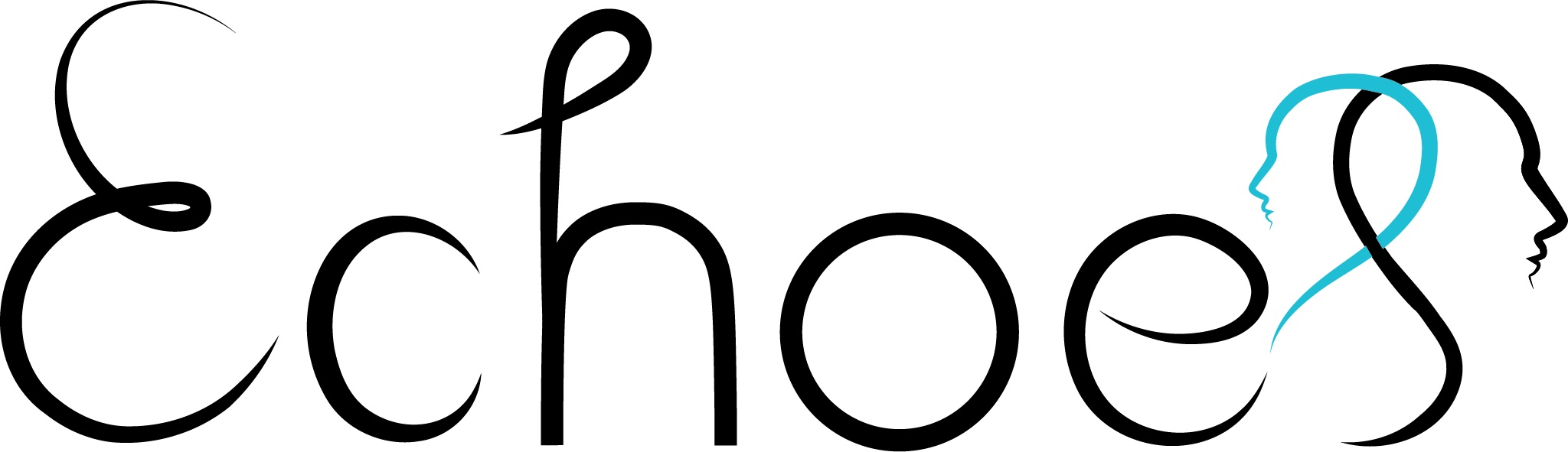


**Chief Investigator: Professor Mark Hayward**

**STUDENT INFORMATION**

**BOOKLET**

(for participation in Phases 2 and 3)

**Support for young people who are distressed by hearing voices:**

Preliminary evaluation of a psychological intervention package for delivery through Mental Health Support Teams within secondary schools (the ECHOES study)


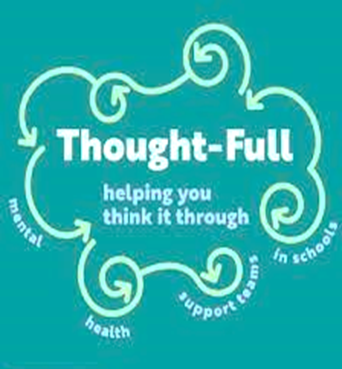


**What is this all about?**

We know that many young people hear voices when no-one seems to be physically present. We want to help young people who are upset by this experience.

We are doing research so we can learn how to offer help to these young people within schools.

**What is research?**

Research is how we try to find out the answers to important questions!

**What will happen in this research?**

- You will meet with a researcher at your school who will ask some questions about your voice hearing experiences and how you are feeling. They will ask the same questions at the beginning and end of the study.
- You will meet with a therapist within school who will help you to develop some strategies for coping with the voices.
- You will also be able to nominate someone you trust (we call them a Plus-1) to attend a workshop where they can learn about hearing voices. They can use the lessons they learn to help you to cope with the voices.

**Why have I been asked to take part?**

We are asking students within Years 7-11 at your school to take part.

We are asking students to take part if they can sometimes be upset by hearing voices.

**Do I have to take part?**

**No**. It is up to you and your parent(s)/carer(s) if you would like to take part.

If you decide not to take part you can still get some help with your emotional problems.

**What do I do now?**

If you do want to take part, you can read the information on the next pages.

**
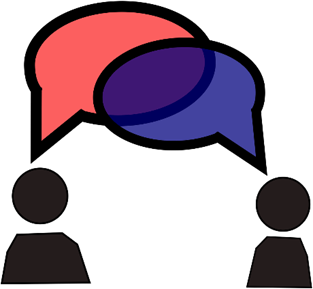
**

You can talk to your parent(s)/carer(s) about the study. They will need to give permission for you to take part. If you do not want to take part then you can stop here.

**A CLOSER LOOK AT THE STUDY**

**MORE INFORMATION ABOUT THE STUDY**

**What is this study about?**

This study is looking at the best ways to help young people who are upset by hearing voices.

Hearing voices is a common experience for young people.

We have developed some helpful interventions within Child & Adolescent Mental Health Services (CAMHS). We want to offer these interventions within schools so young people can get help as soon as possible.

Within the study, there will be three different interventions:

- For you - up to four 1:1 meetings with a therapist from Thought-Full (who provide NHS mental health services within your school)
- For your Plus-1 (this could be a parent/carer, another family member, a friend, etc. – but they must be at least 16 years old and no longer attending school) – a one-off workshop.
- For school staff – a separate one-off workshop

**What will happen during the interventions?**

**1:1 meetings with a therapist**

You will meet with a therapist at school (although you may meet somewhere else or online if you choose to meet during the holidays).

- There will be a maximum of four meetings with the same therapist, usually about a week apart.
- You will talk about the things that can trigger the voices and how you currently cope with them.
- You and the therapist will agree an action plan for trying to use your coping strategies a bit differently.
- You will try your best to put the action plan into practice between the meetings and see if this helps.

**Workshops for the Plus-1 you have nominated**

- You may wish to speak with your parent(s)/carer(s) about who to nominate as your Plus-1.
- You can ask the Plus-1 if they would like to take part in the study. If they agree, we will invite them to attend a one-off workshop (either in-person or online). This person can choose whether or not to attend.
- If this person attends the workshop, they will learn about voice hearing experiences and how to help a young person to cope with the voices.
- We will not talk about you and your personal experiences at the workshop.

**Workshop for school staff**

- We will invite members of the school staff to attend their own workshop.
- The content of the workshop will be the same as the workshop for trusted people.

**What will happen after the interventions?**

- **
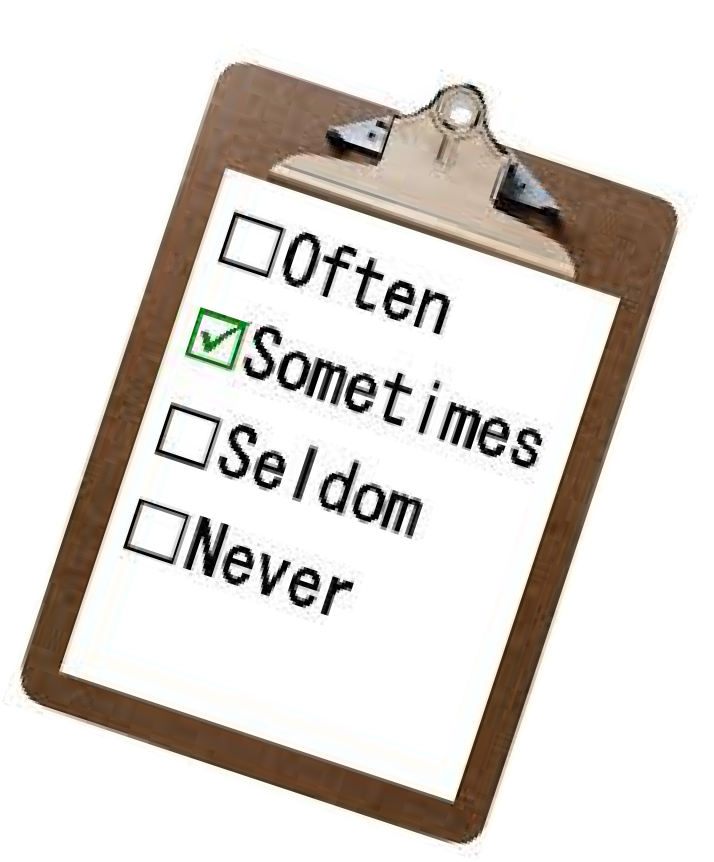
**After the 1:1 meetings have finished, you will meet with a researcher to answer the same questions that you answered at the beginning of the study.
- You will also be invited to offer some feedback on your experiences within the study. This interview will be audio recorded. The recording will be securely stored prior to being transcribed. The transcription will also be securely stored and will contain no identifying information.
- The Plus-1s and school staff who attend the workshops will also be asked some questions and offer some feedback. You will not be involved with any of these processes.
- Your involvement with the study will last for approximately 3 months.

**What are the good things about taking part?**

- By taking part, you will help us to find the best ways of helping young people who are upset by hearing voices.

**Will I get any money for taking part?**

- You will receive a £40 voucher for giving up your time to answer questions at the beginning and end of the study (£20 at the beginning and £20 at the end).

**Are there any bad things about taking part?**

- Some of the questions asked by the researcher at the beginning and end of the study may be upsetting. If this upset persists, the researcher will support you to access some help.
- When you talk about your voice hearing experiences within the 1:1 meetings this may be upsetting. The therapist will help you to cope with this upset and will arrange any support required from school staff.
- You can take a break from or leave a 1:1 meeting at any time.

**Has anyone checked if this research is okay?**

The research has been checked by a group of people called an Ethics Committee. They make sure the research is okay to do

This research was checked by the (???) NHS Research Ethics Committee and they are happy for it to go ahead.

**How will we use information about you?**

- We will need to use information from you for this research study.
-
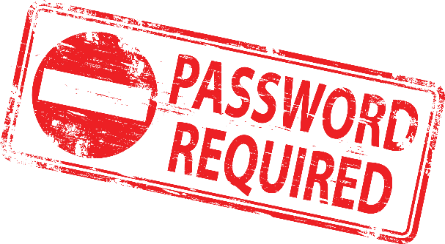
This information will include the name and contact details for you. People will use this information to do the research or to check your records to make sure the research is being done properly.
- People who do not need to know who you are will not be able to see the names or contact details. Your data will have a code number instead.
- We will keep all information about you safe and secure.
- Once we have finished the study, we will keep some of the data so we can check the results. We will write our reports in a way that no-one can work out that you took part in the study.

**What are your choices about how your information is used?**

- You can stop being part of the study at any time, without giving a reason, but we will keep information that we already have.
- We need to manage our records in specific ways for the research to be reliable. This means that we won’t be able to let you see or change the data that we hold about you.

**IMPORTANT INFORMATION**

**What else do I need to know?**

- You **do not** have to take part.
- If you decide not to take part you can still get help from Thought-Full for any emotional problems you may experience.
- You **can stop taking part at any time**. You don’t have to give a reason.

**What if I have questions?**

- If you have any questions you can talk to the researcher or your parent(s)/carer(s).
- Your parent(s)/carer(s) can then contact us by email or phone and we will answer the questions.


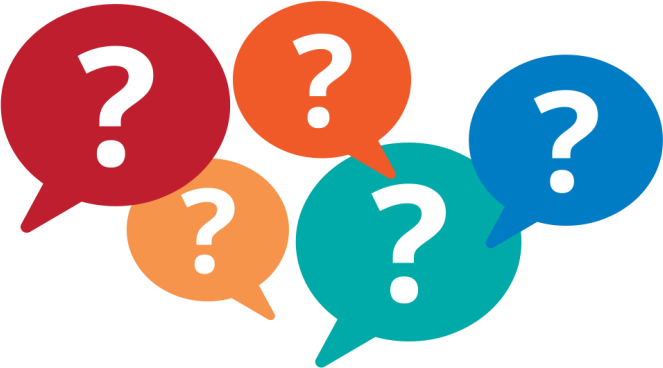


**WHAT HAPPENS NOW?**

If you do want to take part we will ask you to complete a form. This form will give us permission to approach your parent(s)/carer(s).

We will ask you parent(s)/carer(s) for their permission for you to take part.


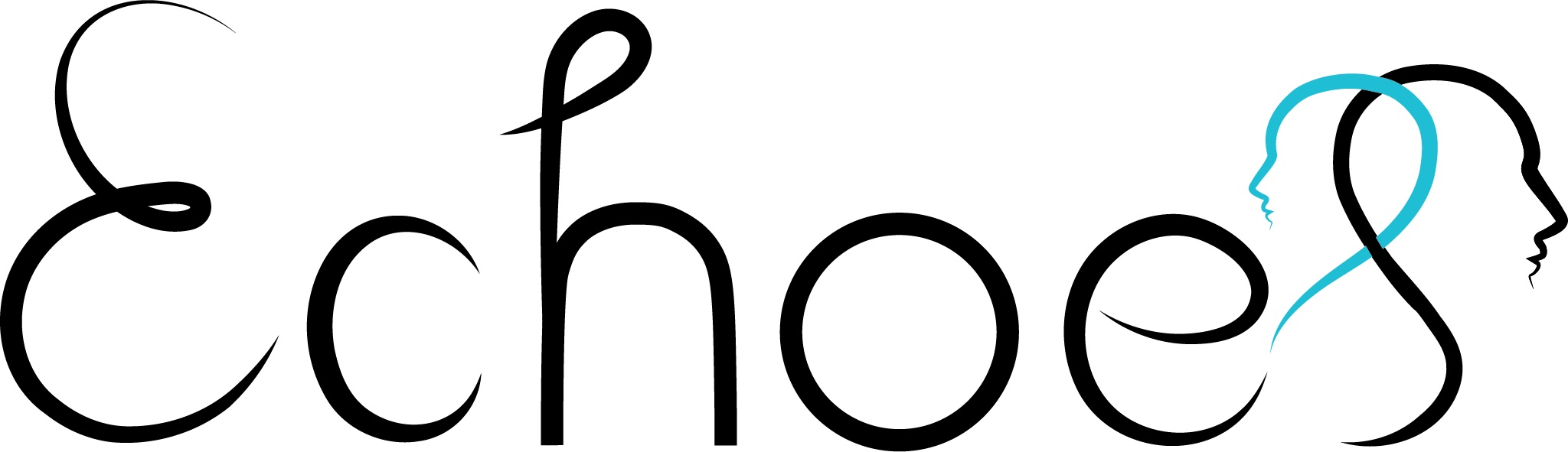


THANK YOU!

For reading this information about our study!
